# Reinforcement-Learning-Based Routing and Resource Management for Internet of Things Environments: Theoretical Perspective and Challenges

**DOI:** 10.3390/s23198263

**Published:** 2023-10-06

**Authors:** Arslan Musaddiq, Tobias Olsson, Fredrik Ahlgren

**Affiliations:** Department of Computer Science and Media Technology, Linnaeus University, 39182 Kalmar, Sweden; tobias.ohlsson@lnu.se (T.O.); fredrik.ahlgren@lnu.se (F.A.)

**Keywords:** Internet of Things, machine learning, reinforcement learning, resource management

## Abstract

Internet of Things (IoT) devices are increasingly popular due to their wide array of application domains. In IoT networks, sensor nodes are often connected in the form of a mesh topology and deployed in large numbers. Managing these resource-constrained small devices is complex and can lead to high system costs. A number of standardized protocols have been developed to handle the operation of these devices. For example, in the network layer, these small devices cannot run traditional routing mechanisms that require large computing powers and overheads. Instead, routing protocols specifically designed for IoT devices, such as the routing protocol for low-power and lossy networks, provide a more suitable and simple routing mechanism. However, they incur high overheads as the network expands. Meanwhile, reinforcement learning (RL) has proven to be one of the most effective solutions for decision making. RL holds significant potential for its application in IoT device’s communication-related decision making, with the goal of improving performance. In this paper, we explore RL’s potential in IoT devices and discuss a theoretical framework in the context of network layers to stimulate further research. The open issues and challenges are analyzed and discussed in the context of RL and IoT networks for further study.

## 1. Introduction

### 1.1. Context

The use of Internet of Things (IoT) devices has increased tremendously, and each year, increasingly more users are utilizing IoT-based solutions for personal [1], financial [2], and commercial applications [3]. The IoT networking paradigm provides communication between devices to connect our physical world [4]. The IoT is a network of smart devices operating in a lossy environment to enable connectivity between things, people, or services. These IoT devices are often battery-powered and contain limited computational capabilities. These resource-constrained sensor devices operate in a lossy environment and are often deployed in harsh environments [5]. IoT application areas span from the smart grid [6], smart transportation [7], and smart industries [8], to smart homes [9]. IoT-based applications can lead to a true realization of smart city environments. This requires a massive number of efficiently deployed IoT devices. The enormous use of IoT-based devices has created a complex network infrastructure generating large amounts of data. The increases in network size and data volume lead to poor network performance. Since these devices are battery-powered, they are therefore referred to as low-power and lossy networks (LLN) devices [10]. Apart from their low power, these devices are not equipped with adequate processing capabilities. Deploying nodes is also challenging and costly due to the complexity and variability of IoT environments. In a complex networking environment, maintaining information and resources is becoming challenging amid growing IoT services [11].

These devices’ communication mechanisms are managed by lightweight protocols specially designed for low-power and lossy devices. For example, in the medium access control (MAC) layer, some IoT devices use an IEEE 802.15.4-based MAC mechanism, which slightly differs in operation compared to the standard IEEE 802.11 MAC protocol [12,13]. The IEEE 802.15.4 standard is a fundamental building block designed for devices with limited access to power sources and limited processing capabilities.

Similarly, the network layer plays a vital role in the architecture of IoT devices. One key protocol designed for IoT networks is the routing protocol for low-power and lossy networks (RPL) [14]. The RPL is also specifically tailored to address the unique challenges of IoT devices. The network created using the RPL protocol is based on a destination-oriented directed acyclic graph (DODAG). The RPL creates a DODAG using a number of control overheads. The periodicity of these control messages is managed by a trickle timer mechanism. The IEEE 802.15.4 MAC and RPL networks based on the trickle timer mechanism are standardized protocols for LLN devices [15]. IoT devices often struggle to manage their limited computation and energy resources using standardized protocols. For example, backoff exponent (BE) is a parameter used in IEEE 802.15.4 and IEEE 802.11 standards, but it functions slightly differently in each standard. In the IEEE 802.15.4 MAC layer, the BE is incremented by one each time the channel is found to be busy, leading to an increase in the BE before frame transmission. A larger BE means the devices wait longer before attempting to transmit. Thus, if the network density or transmission rate increases, the blind increase in BE causes performance degradation [16]. Similarly, in the network layer, the transmission and reception of control overheads consume valuable resources, particularly if the network size increases [17]. To enhance the capabilities of an IoT network, an intelligent DODAG construction mechanism could be employed in the RPL, avoiding the introduction of high control overheads.

### 1.2. Motivation

These resource management challenges have motivated the research community to look for efficient ways to manage the operation of IoT devices. Thus, this opens up new opportunities for the machine learning (ML) paradigm. ML models can enable the classification, detection, and prediction of future events [18]. With such models, resource sharing [19], load balancing [20], channel access [21], and routing can be performed efficiently [22]. Recently, ML, particularly reinforcement learning (RL), has shown promising solutions in the field of network security [23], computer vision [24], natural language processing [25], cognitive radios (CRs) [26], 6G communication [27], and so on. RL is a powerful tool that provides machines with the ability to perform a specific action without being programmed. In RL, an agent learns how to perform actions within an environment to achieve certain goals by receiving feedback in the form of a reward [28].

RL-based techniques have achieved remarkable success in various domains during the past few years. RL algorithms are able to utilize learning processes to adapt to changing environments and make intelligent, long-term decisions. For example, RL has been studied for wireless networking, particularly to enhance specific networking layers [29]. Similarly, an RL-based model for a workflow scheduling framework for edge-cloud environments was proposed to reduce congestion and execution delays [30]. RL-based algorithms have also shown their potential in fog computing environments [31]. In addition, the integration of deep learning with RL (DRL) has demonstrated an improved performance in complex control tasks [32]. DRL has also been applied for trajectory planning for the unmanned-aerial-vehicle-assisted IoT [33]. The RL algorithm is also utilized to simplify the NP-hard optimization resource management problem, demonstrating its effectiveness in optimizing renewable energy use within long-range (LoRa) networks [34]. RL-based approaches have shown their potential to enhance CR capabilities [35]. In a similar manner, RL-based approaches have been applied to solve the issue of limited licensed spectrum availability. RL algorithms address the challenge of achieving both efficient and fair coexistence between long-term evolution and Wi-Fi technologies [36,37]. Numerous algorithms driven by RL have been suggested to enhance the efficiency of IoT devices, such as a computation offloading scheme for healthcare applications [38], spectrum access [39] for IoT networks, and target localization for IoT sensor selection [40]. The resource management problem is also handled by using RL for efficient networking protocols. These studies present a viable option for effectively managing resources within IoT devices through the application of the RL framework.

### 1.3. Contributions

This paper presents a comprehensive review of state-of-the-art RL-based techniques to address resource management challenges in an IoT networking environment. Additionally, this paper also discusses a theoretical framework for applying RL in solving routing problems in IoT devices. IoT and RL-related concepts and the literature review have led to the development of a theoretical framework that aims to increase researchers’ understanding and knowledge for future RL-based studies for IoT devices. The framework also aims to serve as an explanation for RL and IoT device’s decision-making scenarios. In the field of the IoT, we are still at an early stage regarding ML-based devices in general and RL-based IoT networking decisions in particular. Gregor [41] presented their seminal paper on the nature of theory in information systems, and we are at a stage in which we are describing the attributes of RL-based networking and developing taxonomies, i.e., a theory for analyzing and explanation. Numerous research opportunities exist for future development and utilization of design artifacts rooted in robust theories to provide explanations and predictions across various levels of analysis. To highlight prospective areas for future research in the field of RL and the IoT, both areas could significantly benefit from exploring how RL features can be integrated into digital design elements within IoT interfaces.

- We highlight key issues regarding IoT network resource management with a focus on network layer improvements.
- We examine the RL concept as a potential solution to enhance IoT network routing performances.
- A detailed overview of how RL is being applied within the IoT network layer environment is provided.
- We discuss the challenges and explore associated open issues when using RL algorithms in the context of IoT networks.

The remainder of the paper is organized as follows. Section 2 presents the key concepts of the IoT environment, including its system model, network, and MAC layer. Section 3 provides an overview of the fundamentals of RL. Section 4 comprehensively reviews existing works and explains the RL model for IoT networking management. Section 5 discusses the challenges and open issues, followed by Section 6, which concludes the paper.

## 2. Internet of Things Environment

### 2.1. System Model

The end devices in an IoT network consist of a number of IoT sensors generating large volumes of data in irregular patterns. Generally, the IoT network consists of one sink node and a set of N sensor nodes. The IoT network, created using the RPL protocol, splits the N sensor nodes further into a set of child and parent nodes. The network N=PUC, where $P=\{p_{1},\cdots ,p_{x}\}$ is the set of parent nodes and $C=\{c_{1},\cdots ,c_{x}\}$ are the child nodes. Each sensor node participates in generating the data packet at random intervals. All the generated data are directed toward the root node. The parent–child topology creates a network graph, i.e., a DODAG. The node’s position in the network graph is defined with the term ‘rank’. Rank is used to assign a numerical value to each node in the network. These numerical values represent the distance (or cost) of the node from the root node. The rank serves as an indication of the node’s position within the network hierarchy. Rank is measured with a specific objective function (OF). The standardized mechanism uses OF zero (OF0) based on hop counts and minimum rank with hysteresis OF (MRHOF), which is based on the expected number of transmission counts (ETX) metric. DODAGs created using a specific OF are maintained by a control message known as a DODAG information object (DIO). IoT nodes operating in a lossy environment can have fluctuating traffic patterns. In the IEEE 802.15.4-based MAC layer for medium access, channel sensing occurs only once at the end of the BE period, and the BE increases each time the channel is found to be busy.

### 2.2. Network Layer

The network layer based on the RPL mechanism creates routing table entries with the help of three control messages, i.e., the DODAG advertisement object (DAO), DODAG information solicitation (DIS), and the DIO. These messages help to construct the DAG structure. DODAG construction begins with the initiation of the sink node, which broadcasts the DIO messages to propagate information about the network’s structure and configuration, including rank information. The rank information in DIO messages helps nodes assess their position in the network topology. Upon receiving the DIO message, the neighboring nodes measure their rank and broadcast the information to neighboring nodes. This process continues until all nodes within the DODAG have received the DIO message.

**Figure 1:**
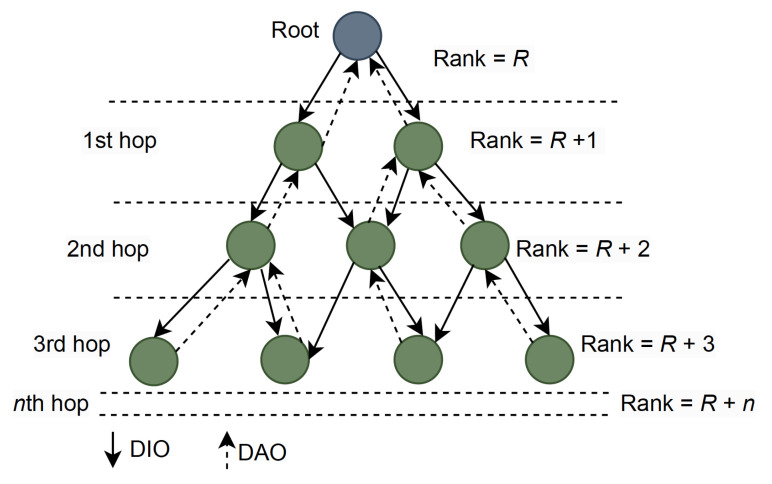
RPL network model with a control message sequence.

DAO messages are used by non-root nodes to build reverse routes, allowing parent nodes to track the routes to their child nodes. If a node does not receive the DIO message, it can request to join the DODAG network by sending a DIS message. These messages help the node to discover its neighbors and help ensure the scalability of the DODAG to allow seamless integration of new nodes into the network. An RPL network model with a control message sequence is illustrated in Figure 1.

The OF defines how nodes translate specific metrics, such as the link quality, energy consumption, or latency, into a value known as rank. Rank is represented as follows [42]:

(1) $$R_{C}=h+R_{P}+R_{increase}$$

where *h* represents the one hop distance defined in RFC 6719 [43]. The $R_{C}$ is the child node rank, and $R_{P}$ is its preferred parent node rank. $R_{increase}$ refers to the numerical value added to a node’s current rank when it becomes a parent for another node in the network. For example, it is the ETX metric for the standardized MRHOF. Since IoT nodes are limited in computational and energy resources, limiting the control overheads is essential. The RPL uses a trickle timer mechanism to control the frequency of DIO messages. It increases the transmission frequency of DIO messages exponentially if the network is unstable. The transmission frequency is reduced to the initial level if the network is stable. The trickle algorithm is based on three key variables, i.e., a consistency counter referred to as counter, the length of the trickle interval denoted as *I*, and a random interval of length *t*. It also has two configuration parameters, i.e., minimum interval length $I_{min}$ and maximum interval length $I_{max}$, to determine the range within which the transmission intervals can vary. Each node also maintains a trickle timer state using the current interval *I* and the number of messages received within that interval *k*. Initially, the value of *I* is set within the range of [$I_{min}$, $I_{max}$], and counter is initialized to 0. Subsequently, a transmission interval *t* is selected from the interval [I/2, *I*]. When a node receives a DIO message, it first checks the message consistency. In the event of consistent transmission, which means no change in the rank status of the node, counter is incremented by 1. The DIO is transmitted if counter is below *k*; otherwise, transmission is suppressed. After the expiration of *I*, the trickle algorithm doubles the interval length until it reaches $I_{max}$. In the case where an inconsistent transmission is received, the timer is reset to the initial value [44]. Thus, by using the trickle timer algorithm, RPL nodes dynamically adjust their transmission intervals based on the stability of the network.

### 2.3. MAC Layer

The IEEE 802.15.4 MAC layer operates slightly differently compared to the IEEE 802.11 access mechanism. The 802.11 MAC protocol operates based on the distributed coordinated function (DCF) mechanism, which first senses the channel and then waits for random backoff if the channel is found busy. If the channel is idle, it waits for a period known as the DCF interframe space (DIFS). After the DIFS period, it randomly selects a contention window (CW) size from 0 to $2^{BE}$ − 1. The value of BE increases by one each time there is a collision [45]. Similar to the 802.11 MAC, IEEE 802.15.4 has three variables, i.e., a BE, the number of backoffs (NB), and a CW. The initial value of BE is set to 3 and can increase up to a maximum of five backoff stages. Transmission starts if the channel is free. Otherwise, the node takes a deferred approach and increases the BE period by one prior to frame transmission [46]. Incrementing the BE during channel sensing is an energy-efficient approach; however, this approach performs poorly as the network size increases. If the BE is increased and no collision is detected after frame transmission, this may add an unnecessary access delay, resulting in network performance degradation.

### 2.4. Resource Management Problems in IoT Environments

A significant characteristic of ubiquitous IoT devices is their constrained resources. IoT devices contain limited resources such as low power and limited memory and processing capabilities. The deployment of IoT devices encounters a number of limitations at the node, network, and application levels. For example, at the network level, one of the major problems in IoT RPL routing is the absence of a proper mechanism for network topology discovery. This can lead to suboptimal routing and decreased network performance. The IEEE 802.15.4 MAC layer also faces significant resource management problems. These limited resources pose major challenges when it comes to network scalability.

IoT devices in a large-scale network are generally heterogeneous in terms of computational power, storage capacity, and communication capabilities. In many practical applications, the complex heterogeneity of the IoT network is a critical challenge for the devices to accomplish the aims of enhancing performance. For instance, OFs are employed within the RPL to enhance particular network metrics, such as energy utilization, delay, and network throughput.

One of the commonly used OFs is the ETX, which provides an estimate of the number of transmission attempts required to successfully deliver a packet between nodes in a network. In a heterogeneous network, where the traffic transmission rate is also heterogeneous, as the network size grows, the computation and maintenance of ETX values for all nodes in the network become more complex and computationally expensive. The ETX metric is determined based on the reception ratio of acknowledgments (ACKs) received from the destination node for the data packets transmitted over the link. The calculation of ETX relies on regular data packet transmissions and the observation of ACKs. When a source node sends a data packet to the destination node, it waits for an ACK to confirm successful reception. If the destination node successfully receives the packet, it returns an ACK to the source node. ETX is sensitive to network conditions, including interference, congestion, and dynamic changes in link quality. Fluctuations in link quality can lead to varying ETX values, making it challenging to maintain stable and consistent routing decisions. ETX is also biased towards shorter paths, since a longer path with more hops requires more transmissions, leading to higher ETX values. This bias can result in suboptimal routing decisions, favoring shorter paths even when longer paths may have a better link quality.

In the MAC layer, increasing the BE during channel sensing is an energy-efficient approach to avoid node collisions. The idea is to increase the waiting time for a node before accessing the shared communication channel, reducing the likelihood of collisions. However, this approach may not perform well in larger networks or under heavy traffic conditions. As the network size or traffic transmission increases, the likelihood of collisions also increases, making it less effective to rely on the BE alone to manage access to the channel. Furthermore, if the BE is increased and no collision is detected after frame transmission, it results in an unnecessary access delay, further degrading network performance.

## 3. Reinforcement Learning

### 3.1. Basic Concept

RL is a type of ML that focuses on learning the environment to maximize the cumulative reward [47]. RL can be broadly classified into two main approaches, i.e., model-based and model-free RL [48]. In a model-based approach, the agent considers the range of future possibilities to decide the possible action beforehand. AlphaZero is one of the notable examples of a model-based approach [49]. In a model-based approach, the agent’s model based solely on its experiences can create challenges such as bias, leading to subpar performance in a real environment. In addition, this approach is very computationally intensive, which can lead to failures. In contrast, model-free methods are not based on “models” and thus are easier to implement and tune, leading to a lower sample efficiency. Model-free approaches can be split into two types based on the learning they are designed to perform. The first is policy optimization and the second is Q-learning. Advantage actor–critic and asynchronous advantage actor–critic [50], as well as proximal policy optimization [51], are prime examples of this optimization method. In Q-learning, an optimal function $Q^{*}\left(s,a\right)$ is learned by approximating it using Q(s,a). Most optimization techniques either use policy optimization or the Q-learning method. However, some algorithms such as [52] use both methods.

**Figure 2:**
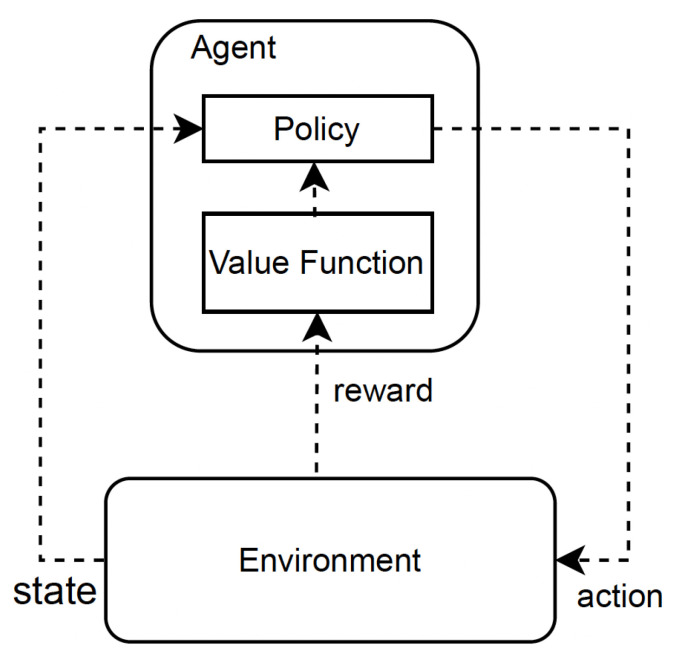
The fundamental operation of the RL mechanism.

In the standard RL mechanism, at each time step *t*, the agent takes action $a_{t}$ based on its current state $s_{t}$ [53]. This action leads to a change in the environment’s state, transitioning from the current state to a new state. The agent then receives a reward $r_{t}$ from the environment, which informs us about the quality of the current state. The agent’s ultimate goal is typically defined in terms of maximizing the cumulative rewards over time. Thus, the RL algorithm provides a way for the agent to learn the optimal behavior that leads to achieving its goal [54]. The common symbols used in RL frameworks are described in Table 1. Figure 2 depicts the fundamental operation of an RL framework.

### 3.2. State Space

The training of the RL system involves learning from trial and error by interacting with the dynamic environment. The state of the environment plays a crucial role in determining the action taken by the agent. RL models use a state–action pair or an estimated value function that represents the desirability of the current state. In most environments, the state transition follows the Markov property, meaning that the current state $s_{t}$ provides sufficient information to make an optimal decision [55]. The model containing state, action, reward, and state transition T is referred to as the Markov decision process (MDP). The MDP is a tuple of $\langle S,A,T,R\rangle$, in which *S* is the set of all possible states, *A* is the set of possible actions, *T* is the transition function, and *R* is the reward function. The system is said to be Markovian if the future state of the environment depends only on the current state and the action taken in that state and it is independent of the sequence of states that preceded it.

### 3.3. Action Space

The action space refers to the set of all possible actions that an agent can take in a given environment. The agent’s decisions are completely dependent on the environment in which it operates. Thus, different environments result in different action spaces [56]. In some environments, such as Atari and Go, the action space is discrete, meaning that only a finite number of actions are available to the agent [57]. In these cases, the agent must choose one of the available actions at each step. On the other hand, in other environments, such as controlling a robot in a physical world, the action space is continuous [58]. This means the agent can choose an action from a continuous range of values rather than a limited set of options.

### 3.4. Reward Function

The reward function, $r(s_{t},a_{t})$, represents the value of taking a particular action, $a_{t}$, in a given state, $s_{t}$. The goal of the agent is to determine the best policy that maximizes the total reward. The reward function specifies the learning objectives of the agent and is updated at each step based on the new state and action taken.

### 3.5. Policy

The policy refers to a strategy or a set of rules that an agent employs to determine its actions in various states of an environment. The policy represents the strategy to map the states to actions. A deterministic policy directly maps states to specific actions, where it provides a probability distribution over actions. The policy is often represented by π(a|s), where *a* is an action and *s* is a state. The optimal policy, $\pi^{*}$, is the one that maximizes the expected cumulative reward received by the agent.

### 3.6. State Value and State–Action Value Function

The state value function, denoted as $V^{\pi }\left(s\right)$, is used to specify the long-term desirability of being in a specific state. On the other hand, the state–action value function, referred to as the Q-function ($Q^{\pi }\left(s,a\right)$), specifies how good it is for an agent to take certain action *a* in state *s* under a given policy π. In Q-learning, the Q-values of state *s* and action *a*, i.e., Q(s,a), is determined as follows [59]:

(2) $$Q^{\pi }\left(s_{t},a_{t}\right)=E_{\pi }\left[r_{t+1}+\beta \max_{a^{\prime }}Q^{\pi }\left(s^{\prime },a^{\prime }\right)|s_{t}=s,a_{t}=a\right]$$

Equation (2) is referred to as Bellman’s equation, in which $Q^{\pi }\left(s,a\right)$ is the Q-value for state *s* and action *a* under policy π, $E_{\pi }$ is the expected value under policy π, $r_{t+1}$ is the immediate reward received, β is the discount factor, $s^{\prime }$ and $a^{\prime }$ are the next state and action, respectively, and $s_{t}=s$ and $a_{t}=a$ are the current state and action.

In addition, we obtain the expected discounted returns for the next potential state–action pair. The update rule for the Q-value function is described as follows:

(3) $$Q(s_{t},a_{t})=(1-\alpha )\cdot Q(s_{t},a_{t})+\alpha (R(s_{t},a_{t})+\beta \cdot \sum_{a^{\prime }}\pi (a^{\prime }|s_{t+1})\cdot Q(s_{t+1},a^{\prime })-Q(s_{t},a_{t}))$$

The values of α and β are between 0 and 1. The learning rate α indicates to what extent new information overrides the previous information. If α is 0, the agent learns nothing and relies on previous knowledge only, whereas if α is 1, the agent only considers new information irrespective of previous knowledge. Similarly, the discount factor β indicates the importance of future rewards. If β is 0, that means only the current reward is considered, and if β is 1, the agent considers long-term future rewards. The estimated Q-values are stored in a look-up table for each *s* and *a* pair. The update rule adjusts the current Q-value estimate based on the observed reward and the maximum expected cumulative reward from the next state. This allows the Q-value estimate to converge towards the true Q-value as more and more experience is gathered.

## 4. Reinforcement Learning for IoT Networking Management

This section presents an overview of the key algorithms that use RL to handle resource management issues in an IoT environment. The focus of this review is to explain how RL-based solutions in the IoT environment formulate the state space, action space, MDP, and reward. This section also discusses the theoretical framework for applying the RL model for RPL management.

### 4.1. Related Research Studies

Routing management is crucial for decentralized and autonomous networks, particularly for networks with limited resources. In addition, wireless networks are affected by various factors, such as signal interference, signal propagation, or network traffic. All such factors impact network communication and connectivity. The changing network dynamics in the wireless environment affect decisions related to routing or network management. In traditional routing, each device should learn and adapt its routing policy to handle the varying network conditions. Conventional routing protocols rely heavily on rule-based decisions that cannot adapt to the dynamic nature of network environments. Such protocols cannot make performance-based routing decisions that can optimize network throughput and reduce latency. As a result, there is a growing research focus on developing intelligent decision-making strategies that are context-aware and employ RL techniques. These techniques have the potential to enhance network performance in the presence of environmental fluctuations and other uncertainties.

Numerous studies are present in the literature that have adopted RL for efficient routing decisions. For example, Ref. [60] proposed RL-based congestion-avoided routing (RCAR) for underwater acoustic sensor networks to reduce end-to-end delays and energy consumption. The RL mechanism in RCAR is based on the status of the buffer size, battery, and neighboring node locations. In a similar context, Ref. [61] presents a distributed RL-based protocol called CARMA for channel-aware RL-based multi-path adaptive routing, which facilitates next-hop selection for a node based on the number of unsuccessful transmissions. This approach enables efficient routing decision making by dynamically adapting to the evolving network conditions, thus improving the overall network performance. Similarly, RL was applied in routing for CR-enabled IoT communication [62]. This approach incorporates channel selection decisions with routing decisions at the network layer to provide improvements to the average data rate and throughput. Another approach proposed by Mao et al. [63] introduced a solution for routing in software-defined networks (SDNs) that leverages convolutional neural networks (CNNs) for periodic learning of network dynamics. This approach enables the network to continuously adapt and optimize its routing decisions based on network conditions.

The authors of [64] explore RL of routing in CR ad hoc networks with the aim to reduce protocol overheads and end-to-end delays and improve the packet delivery ratio. Stampa et al. [65] proposed a DRL approach for optimizing routing in SDNs. The agent in this approach adapts to traffic conditions to minimize network delays. The proposed method is able to effectively learn the underlying network dynamics and optimize the routing policy accordingly, resulting in an improved network performance. Similarly, the authors of [66] proposed a machine-learning-assisted centralized link-state routing system for an SDN-based network. This paper explores a routing algorithm called MLaR that makes real-time routing decisions based on historical network parameters such as the latency, bandwidth, signal-to-noise ratio, and distance with the help of ML. Their proposed approach highlights the innovative aspect of applying ML to the centralized link-state routing algorithm.

Cheng et al. [67] introduced a Q-learning-based adaptive zone partition (QAZP) approach. In this method, an agent within a mobile anchor node is equipped with a directional antenna to divide the network into distinct zones corresponding to individual sinks. The zone size is adjusted to balance the power consumption, leveraging the remaining energy of sensor nodes situated in proximity to each sink. In [68], a Q-learning-based approach has been developed for task modeling in dynamic wireless sensor networks (WSNs) that focuses primarily on task scheduling for cooperative sensor nodes involved in target tracking. Wei et al. [69] proposed ISVM-Q, an algorithm that combines the Q-learning RL technique with an enhanced supervised learning model. ISVM-Q is designed for optimizing task scheduling within sensor nodes. In [70], a link quality monitoring mechanism based on RL was introduced for the RPL protocol. This approach aims to continuously update the network’s routing information and promptly respond to fluctuations in link quality and changes in topology, which may occur as a result of node mobility. The authors of [71] proposed an approach to enhance the quality of service (QoS) and security of routing in SDN-based IoT environments. The reward function for QoS-aware routing considers parameters such as the end-to-end delay, packet loss rate, and energy consumption. A Q-learning reliable routing approach with a weighting agent (QLRR-WA) was introduced in [72]. The QLRR-WA algorithm aims to optimize network performance by learning a set of weights that minimize the weighted cost equation. The weights are represented as the states of the agents, and the agents take continuous actions to improve their weights. The reward is determined based on the average network latency and expected network lifetime, which encourages the agent to improve the network’s reliability.

The authors of [73] introduced QGeo, an extension to Q-routing designed to incorporate unmanned robot mobility. QGeo employs periodic Hello packets to update the GPS locations of nodes, enabling them to select the next hop based on the geographic distance to the destination. As a result, the distance metric serves as the primary guide for routing decisions. This approach enables more efficient and effective routing in scenarios where unmanned robots are utilized, thereby contributing to the advancement of robotic systems. In [74], Sharma et al. proposed tailored Q-learning in WSNs to optimize the routing efficiency by minimizing the energy consumption in sensor nodes through a modified Q-learning technique. This technique explores alternative routes through local information sharing. Sink nodes act as agents, broadcasting messages to other sensor nodes, allowing them to iteratively learn and build their routing tables based on these messages.

The authors of [75] also propose a new routing approach for WSNs that aims to improve energy efficiency during information transmission. This approach allows nodes to dynamically select optimal neighboring nodes for energy-efficient transmission. In this approach, the sensor nodes gather and analyze different parameters related to neighboring nodes, such as their transmission direction, distance, and energy consumption. This information is used to update the Q-values of the neighboring sensors, which enables the wireless sensor to determine the most suitable neighboring sensor to transmit information based on the Q-value. Akbari and Tabatabaei [76] also present a routing mechanism that utilizes fuzzy logic and RL to determine optimal routes based on sensor nodes’ remaining energy, bandwidth, and distance to the sink. The proposed approach prioritizes maximizing the lifetime of sensor networks.

The authors of [77] proposed a routing algorithm for mesh IoT networks. The authors aimed to enhance the energy efficiency of the routing approach by introducing a cost function based on the transmission power and remaining energy of both the transmitting and receiving nodes. Another RL-based technique called RLProph [78] was proposed to treat the opportunistic environment as an MDP and apply a dynamic programming-based iterative algorithm to enhance delivery performance. The study in [79] introduces a routing agent that utilizes Q-learning to adjust the routing policy based on local information, aiming to achieve an optimal solution that balances network latency and lifetime. The proposed agent is rewarded for actions that extend the network lifetime and decrease the average network latency. The work in [80] proposes a multi-hop routing technique for QoS optimization in LoRa networks using the RL mechanism. This approach optimizes transmission policy parameters such as the spreading factor, bandwidth, code rate, and carrier frequency to achieve a high QoS in LoRa communication. Kaur et al. [81] also proposed a DRL approach to enhance the routing scheme for IoT-enabled networks. The proposed DRL-based intelligent routing scheme reduces delay and improves the overall network lifetime. The authors suggested a novel clustering method that aims to prevent energy imbalances within the network. This scheme utilizes unequal clustering, multiobjective optimization, and load balancing to enhance the network performance and lifetime. In [82], the authors introduced a multi-hop state-aware routing approach based on traffic flow predictions. This strategy employs recurrent neural networks in conjunction with a deep deterministic policy gradient technique.

Krishnan et al. in [83] proposed a model that aims to avoid energy hole issues and inefficient data collection to preserve network stability while improving the routing performance using a Q-learning approach. The authors of [84] present a new adaptive routing protocol called AQ-Routing, which is based on RL to handle mobile ad hoc network (MANET) IoT systems. The AQ-Routing technique can detect each node’s mobility level in the network. The mobility detection model allows each node to adjust its routing behavior based on the updated mobility factor. Pandey et al. in [85] addressed the issue of establishing extensive connectivity among IoT devices spanning a wide geographic region. The paper proposes an RL-based technique to address multi-hop data transmission challenges such as higher latency, increased interference, and reduced throughput. The proposed approach periodically updates the network’s Q-matrix and makes relay device selections at discrete time intervals to optimize the cumulative reward value for chosen device gateway pairs.

The authors of [86] proposed MeFi to address the challenges of maximizing the energy efficiency and network lifetime of battery-powered sensor networks. MeFi is based on the mean-field RL mechanism and considers the average behavior of the network nodes instead of dealing with each node individually. The authors leverage mean field theory to manage the vast state space caused by numerous devices by focusing on interactions among neighbors. The authors also introduce a prioritized sampling loop-free algorithm to prevent routing loops and discard suboptimal routing strategies. In [87], the authors proposed a routing scheme for Internet of Medical Things networks. The proposed scheme categorizes network traffic into three classes, customizing the QoS for each, and divides the network into zones to reduce message exchanges. Table 2 presents a summary of related work in the field, highlighting their contributions, application domains, and the algorithmic models they employed.

### 4.2. RL Model for RPL Management

**Figure 3:**
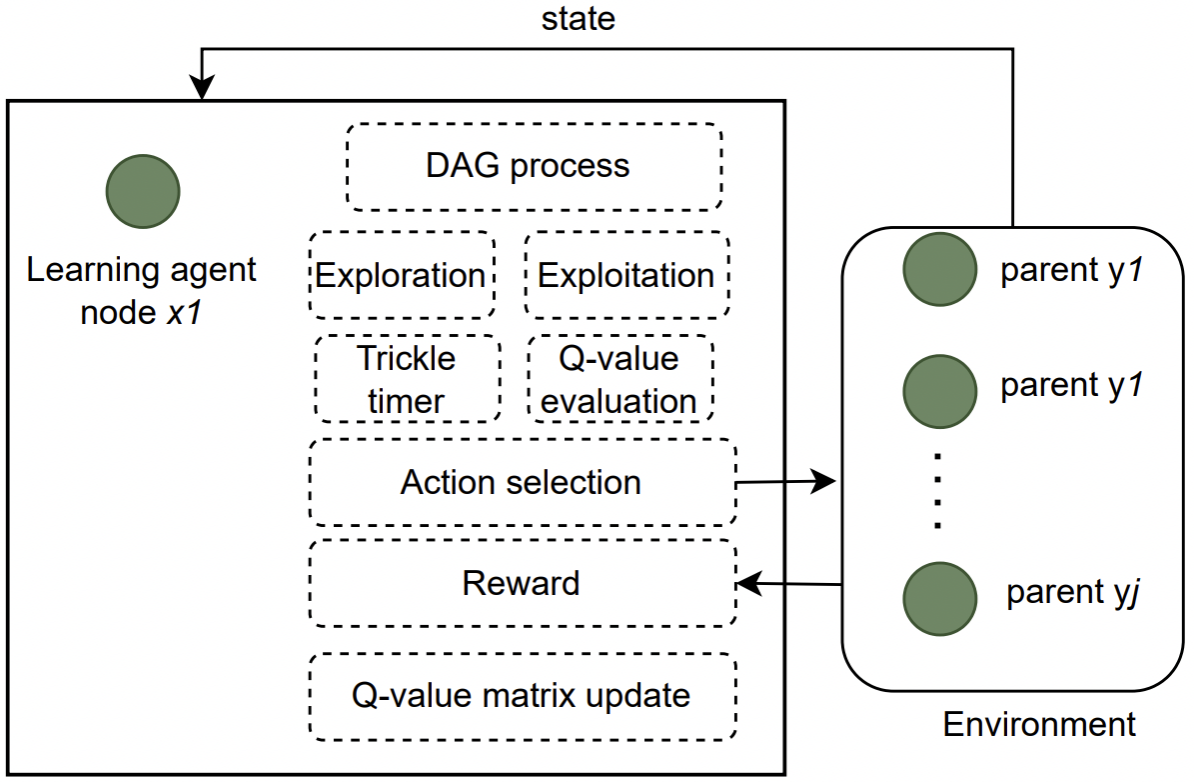
Illustration of RL-based RPL routing.

We can observe from the previous section that devices such as IoT nodes can learn certain policies to improve the network performance. This section describes how the RPL-based network layer described in Section 2.2 can be mapped to the RL mechanism. RL-based RPL routing is illustrated in Figure 3. The RPL forms a DAG according to a specific OF. The nodes construct and maintain these DODAGs using DIO messages. DIO messages significantly contribute to the total network control overheads as they are periodically broadcasted to maintain and update the network topology. To conserve scarce resources, the RPL must generate minimum control overheads while maintaining network quality.

(1) State Space: State $s_{t}$$\left(s_{t}\in S\right)$ can be defined as the state observed by the DODAG child at time *t*, which refers to the rank status of the child node in the DAG. Each child node has a set of *i* states, S=(0,1,2,…,i). The selection of the forwarding path in the RPL mechanism can be based on node rank values. The MRHOF-based RPL mechanism uses ETX for rank calculation. The rank is measured as,

(4) $$Rank\left(x\right)=h\left(x\right)+Rank\left(y\right)+ETX\left(x,y\right)$$

where h(x) is the hop-count of child node *x* towards the root node, Rank(y) is the selected parent *y* rank, and ETX(x,y) is the ETX value between child *x* and parent *y*. The ETX value acts as a reward function for Equation (4). The reward function is described in Equation (12). Based on the reward function, each node updates its rank value during each state transition process. The rank of the root node is Rank(root)=1. Each node broadcasts a DIO message containing its rank value. After receiving DIO messages, the nodes can generate a list of potential parents as,

(5) $$Y_{x}=\left\{n_{x}\in N|h\left(n_{x}\right)<h\left(x\right),ETX\left(x,n_{x}\right)<\delta \right\}$$

where $Y_{x}$ represents the list of potential parents of child node *x*, $n_{x}$ represents the set of all one-hop neighboring nodes of child *x*, and δ is a threshold to remove neighbors with unreliable links. To avoid routing loops, the child selects a forwarding path only if its number of hops from sink nodes is less than its own hop counts $h\left(n_{x}\right)<h\left(x\right)$.

(2) Action Space:A node can select a parent from the list of potential parents. The action $a_{t}$$\left(a_{t}\in A\right)$ is defined as the selection of forwarding parent *y* from the list of potential forwarding parents. Environment *E* is defined as a wireless medium. When the IoT node performs the action $a_{t}$ at the time *t*, the state changes from $s_{t}$ to $s_{t+1}$. The IoT node receives a reward from the environment *E*. A node *x* selects a parent $y_{x}$ from a list of potential parents $Y_{x}$.

In a given time period *t*, a node *x* has some rank value obtained from the last iteration using (5). At a given rank status, the node can select the next parent from the list of a finite number of potential parents $\left(Y_{x}\right)$. Thus, at each state *s*, there is a set of a finite number of permissible actions, which are elements of set *A*.

The state–action space is S×A, where $S=\left(s_{1},s_{2},\ldots ,s_{i}\right)$ and $A_{\left(s\right)}=\left(a_{1},a_{2},\ldots ,a_{j}\right)$. The Q-values are stored in a look-up table for each state–action pair as,

(6) $$\left(\begin{array}{cccc} & y_{1} & \cdots & y_{j}\\ Rank\left(x_{1}\right) & \left[\begin{array}{c} Q_{\left(Rankx_{1},y_{1}\right)}\\ p_{\left(s_{1},a_{1}\right)}^{\pi } \end{array}\right] & \cdots & \left[\begin{array}{c} Q_{\left(Rankx_{1},y_{j}\right)}\\ p_{\left(s_{1},a_{j}\right)}^{\pi } \end{array}\right]\\ Rank\left(x_{2}\right) & \left[\begin{array}{c} Q_{\left(Rankx_{2},y_{1}\right)}\\ p_{\left(s_{2},a_{1}\right)}^{\pi } \end{array}\right] & \cdots & \left[\begin{array}{c} Q_{\left(Rankx_{2},y_{j}\right)}\\ p_{\left(s_{2},a_{j}\right)}^{\pi } \end{array}\right]\\ \vdots & \vdots & \ddots & \vdots \\ Rank\left(x_{i}\right) & \left[\begin{array}{c} Q_{\left(Rankx_{i},y_{1}\right)}\\ p_{\left(s_{i},a_{1}\right)}^{\pi } \end{array}\right] & \cdots & \left[\begin{array}{c} Q_{\left(Rankx_{i},y_{j}\right)}\\ p_{\left(s_{i},a_{j}\right)}^{\pi } \end{array}\right] \end{array}\right)$$

The reward is utilized to update the Q-value matrix $Q(s_{t},a_{t})$ using Equation (2). This current Q-value matrix affects the subsequent action selection. For action selection, we can adopt the ϵ greedy mechanism. In the learning process, the learning agent balances the short-term and long-term gains with exploration and exploitation. The ϵ greedy mechanism performs the exploration using ϵ as a probability parameter. For action selection, a random number *rand* between 0 and 1 is generated, which is then compared with the probability parameter ϵ.

(7) $$a_{t}=\left\{\begin{array}{cc} argmin_{y_{x}\in Y_{x}}\left\{Rank\left(y_{x}\right)\right\}, & rand<\epsilon ,\\ argmin_{a_{i}}Q\left(s_{t},a_{j}\right), & rand\geq \epsilon , \end{array}\right.$$

Exploration is performed if *rand* <ϵ. During the exploration phase, node *x* selects its best alternative parent $y_{x}$ with a minimum rank. If *rand*≥ϵ, the action with the largest return reward will be selected. Let *i* be the index of action from a set of actions *A* and $a_{i}$ represent the *i*-th action in action set *A*.

(3) Reward Function:The core of Equation (4) is the reward function $r(s_{t},a_{t})$. The learning objectives are achieved using appropriate reward functions. With each action $a_{t}$, state $s_{t}$ changes to $s_{t+1}$ and reward function $r(s_{t},a_{t})$ updates to $r(s_{t+1},a_{t+1})$. The reward function *r* of the RPL-based network layer algorithm can be defined with regard to link quality assessment between the child and parent node. The number of link layer retransmission attempts reflects the throughput of an individual link. The ETX measures MAC layer frame transmissions and retransmissions. ETX estimation involves measuring the probability of the frame loss ratio at the link *l* to each neighboring node in both the forward ($d_{f}$) and reverse ($d_{r}$) directions. The probability of unsuccessful frame transmission from node *x* to node *y* is calculated as follows [88]:

(8) $$p=1-\left(1-p_{f}\right)\times \left(1-p_{r}\right)$$

where $p_{f}$ is the probability of a transmission failure and $p_{r}$ is the probability of a reception failure. The ETX for a successful delivery of a frame within a single hop after *k* attempts is measured as:

(9) $$ETX_{l}=\sum_{k=1}^{\infty }k\times p^{k}\times {\left(1-p\right)}^{k-1}=\frac{1}{\left(1-p\right)}$$

The measurement of ETX with respect to the forward delivery ratio $d_{f}$, i.e., $\left(1-p_{f}\right)$, and reverse delivery ratio $d_{r}$, i.e., $\left(1-p_{r}\right)$, is calculated as:

(10) $$ETX_{l}=\frac{1}{\left(d_{f}\times d_{r}\right)}$$

Alternatively, the ETX of the link is the inverse of the probability of successful packet delivery or link reliability, represented as:

(11) $$ETX_{l}=\frac{1}{\;\mathrm{reliability}\;(l)}$$

The reward associated with transmitting from a child node to a parent node is defined as follows:

(12) $$R=\mathrm{sgn}(ETX_{\mathrm{new}}-ETX_{\mathrm{old}})$$

where $ETX_{\mathrm{new}}$ represents the new value of ETX, $ETX_{\mathrm{old}}$ represents the previous value of ETX, and the sign function sgn returns −1 if ETX increases and +1 if the value of ETX decreases.

### 4.3. Federated Learning Model for RPL Management

This section briefly summarizes how the components of federated learning (FL)-based solutions in the IoT environment are formulated. This section highlights the key FL concepts and visions of using FL in IoT networks. FL has the potential to further enhance current IoT systems. It is a particularly attractive solution to build a distributed IoT system due to growing privacy leakage concerns.

FL in IoT networks is composed of two primary components: the data clients, such as IoT devices, and an aggregation server acting as either a base station or an access point. FL allows the IoT devices and a server to train a global model while keeping the raw data in the devices. In FL-based training, each IoT device learns and trains a model using its local dataset. This locally trained model in FL is referred to as a *local model*. After the training, the devices transmit their local model to the server node or base station and then aggregate to create a shared model, which is referred to as a *global model*. This FRL-empowered mechanism can be introduced for LLN optimization. The goal of the FL mechanism is to train a global model. To generate an FL route update model, the IoT nodes can exploit the FL algorithm by which the participating child nodes collaboratively learn a shared model while keeping all the training data locally. Thus, FL is a distributed collaborative approach of IoT devices for data training with a central server node.

The general FL process includes the following key steps. (1) Initialization:The child nodes first set up a learning parameter, such as rank measurement within the network hierarchy. The child nodes can share their rank values with neighboring nodes through the use of a DIO control packet. (2) DistributedLocal Training: The nodes with a similar rank hierarchy form a learning group, and one of the nodes in the group assumes the role of the learning server node, responsible for coordinating the training process. The learning agent, acting as the server node, initiates the training by updating the learning model using a locally learned model from child nodes. Each child node in a group obtains its value function using its own local data, thereby creating a local model. (3) ModelAggregation: After completing local training, the local models are aggregated to create a global model. At this stage, each device has undergone local training using its own local dataset. Each node sends its local model to the server node. After collecting all model updates from local IoT devices, the server aggregates them to calculate the global model. One of the main challenges of model aggregation is the need for efficient communication between the learners and the server node. Transmitting the entire local model from each node to the server node can be impractical due to bandwidth constraints. The learning devices exchange only model parameter updates such as Q-values. The frequency of communication between child nodes and the server node depends on the FL application and network conditions. It can be periodic, event-driven, or adaptive based on factors such as data availability or resource constraints. In addition, synchronization in terms of the timing of model updates is a crucial aspect of FL systems. Inconsistency in updating the local learning models may lead to convergence issues. Convergence criteria can be based on the change in global model parameters.

After federated averaging, the server node uses this aggregated value to obtain its next value function. The learning agent updates the learning model using a locally learned model from other child nodes with the same wireless environment. Model aggregation in this scenario operates iteratively, with multiple rounds of local training and aggregation. In each round, nodes update their local models based on the global model, and these updates are subsequently aggregated. In this way, child nodes computing the forwarding path can learn the wireless environment faster using a global learning model from the learning agent. The child nodes assume a learning agent for global learning of the wireless environment. Individual learned values may suffer from an overestimation of reward information, leading to suboptimal or biased decision making. Each node integrates its learned Q-values in the DIO packet to collaborate with the learning agent. Thus, it provides a second value function for the learning agent. The global learned values in the learning agent represent a fair estimation of the Q-value. In other words, both the local learned values and global learned values are obtained from the same set of experiences in the same environment. In this way, the learned Q-values have a lower chance of error variance.

## 5. Challenges and Open Issues of RL-Based Algorithms in IoT Networks

IoT-based applications have experienced tremendous growth, providing new perspectives on data gathering and transmission. These applications, such as smart and sustainable cities, can potentially affect diverse areas of our lives. Smart city IoT infrastructures span from local area networks to city-wide area networks. These networks include a number of IoT applications, such as smart street lights, smart parking management, smart surveillance systems, and so on. These applications generally depend on the limited resources of IoT devices, particularly energy and efficient communication resources. The RPL protocol can be applied to manage and route data in smart street lights, facilitating real-time control and energy conservation. Similarly, in smart surveillance systems, numerous cameras and sensors are deployed across a city to monitor security. The RPL ensures that the data from these devices are efficiently transmitted to central monitoring stations. RPL-protocol-based routing aided by the RL mechanism can be helpful for such applications to reduce communication overheads and energy consumption. Devices learning to route with minimum overhead support would enable working for longer periods of time. While RL has proven to be a robust methodology to infuse intelligence into IoT devices for communication operations, several issues and challenges still hinder the full exploitation of RL’s potential to aid the IoT paradigm.

This section discusses the critical challenges and corresponding open issues that need to be addressed to employ RL-based approaches to enhance IoT device capabilities. RL-based algorithms require time and resources to process large volumes of data during the exploration and exploitation phases. Meanwhile, IoT device resources are limited in terms of storage, energy, and computation. Running an RL algorithm for a long time on IoT devices is challenging and, therefore, requires a lightweight and appropriate algorithm design to handle such challenges. This section outlines the following primary challenges and investigates the open issues related to employing RL-based methods for IoT device networking.

(1) Synchronization:Devices learning routes through RL-based algorithms such as Q-learning or the policy gradient method can lead to different routing information due to the stochastic nature of the network. The synchronization problem refers to the challenge of ensuring that all devices in the network have consistent and up-to-date information about the optimal routes.

(2) HighDimensions of the State–Action Space in Large Networks: The dimensions of the state–action space can considerably affect the performance of RL-based routing mechanisms. Particularly, if the network size increases, it makes it difficult for an IoT device running the RL algorithm to explore all possible state–action pairs. Such a problem is also referred to as the “curse of dimensionality,” where the number of possible states and actions grows exponentially. Exploring the large number of samples to explore the state–action space causes poor performance and slow convergence.

(3) Accuracyin RL Decision Making: Achieving accuracy in decision-making is one of the critical challenges, especially in scenarios where precise actions are essential. Generally, RL algorithms are concerned with achieving maximum rewards. However, making more optimal decisions, such as in a scenario like autonomous driving, is of utmost importance. Accuracy is also particularly challenging in noisy and uncertain environments. Selecting appropriate metrics for evaluating accuracy in RL is non-trivial. Metrics should align with the specific goals of the RL problem. For example, in the case of IoT networks, the devices may be heterogeneous in nature and often have specific QoS requirements, such as low latency for real-time applications or energy-efficient routing for battery-powered devices. Finding the right balance between these objectives can be challenging, as it requires considering multiple factors simultaneously. Accuracy can be evaluated on a per-episode basis or aggregated over multiple episodes to obtain a more stable estimate of the agent’s overall performance. However, in more complex tasks, accuracy may require a more nuanced definition.

(4) ConvergenceDelay: Convergence is one of the critical factors in the operation of the RL algorithm. The convergence rate of an RL-based routing mechanism depends on the network size. As the number of nodes increases, the convergence rate decreases due to the large number of state–action pairs that need to be explored. In addition, traffic patterns and node mobility also affect the convergence rate. Due to convergence delays, the agent or IoT device would take longer to learn and adjust its routing decision. With longer delays, the agent may continue to transmit packets through a sub-optimal path, leading to a high packet loss rate and lower throughput. Suboptimal path choice may also lead to load balancing issues, leading to instability and congestion in the network. Certain RL-based routing models can handle dynamic link properties, such as delay, reliability, and utilization. In addition, they can manage temporary link cancellations by adjusting the attribute weights to zero or infinity. However, they face significant challenges when the structure or configuration of the network topology changes, such as the addition of new links or nodes to the network. The introduction or removal of nodes from the network results in dimensional changes in the state–action pairs of the RL model. Such a scenario increases the computation overload, training time, and complexities.

(5) DelayedReward: Generally, immediate feedback is often available in an RL mechanism. However, some IoT applications can lead to a delayed or sparse reward that can complicate the operation of the learning process. For example, adjusting a device’s power consumption may not yield instantaneous changes in energy consumption. This delay in receiving the feedback may hinder the agent’s ability to make intelligent decisions. The learning agent making decisions in such environments must account for their actions’ delayed effects. Similarly, the agent must determine the contribution of past actions to the current outcome. Approaches like value iteration, policy gradients, and model-based RL can help agents make informed decisions in such environments. Temporal difference learning and eligibility traces are also commonly used to handle these situations.

(6) HierarchicalLearning: In an IoT network, devices may be organized in hierarchical structures with varying levels of abstraction. This creates a challenge and complexity in coordinating and optimizing actions across different levels of the hierarchy while accounting for local and global objectives. Such structures also introduce temporal dependencies, where actions at one level may have cascading effects on higher levels. RL agents in such scenarios need to be able to learn policies at different hierarchical levels while considering their distinct objectives.

(7) DistributedDecision Making: In a large-scale network, multiple IoT devices can collaborate to optimize a common objective, such as reducing the network delay or energy consumption. In such coordination among agents, handling dynamic network conditions and ensuring convergence to optimal solutions are challenging. Generally, distributed decision making for resource-constrained IoT device networks can be achieved with the help of edge computing to process the data. Distributed decision making may or may not involve learning; the IoT devices can make decisions based on fixed, pre-defined rules.

(8) Multi-AgentLearning: The typical IoT network consists of a large number of IoT devices with heterogeneous sensing, computation, and communication capabilities. Multi-agent learning problems arise due to the distributed and dynamic nature of IoT networks. When dealing with multi-agent learning, the RL algorithm must be able to handle limited device resources in changing network conditions. Multi-agent learning can be applied to develop self-organizing routing algorithms that adapt to changing networking conditions and traffic patterns. Multi-agent systems also face problems such as credit assignments, in which it becomes hard to determine which actions of an agent contribute to a particular outcome. Particularly in a large-scale system, agents must be able to determine not only their own learning objectives but also the potential consequences of their actions on other agents. Similarly, the agents may have conflicting objectives that can lead to suboptimal outcomes. In terms of IoT device routing scenarios, the devices can converge to Nash equilibrium, where no device is incentivized to unilaterally change its routing strategy, especially in a non-convex and dynamic environment. In such scenarios, techniques from game theory can be employed to analyze and identify a Nash equilibrium.

(9) Real-timeResponsiveness: Some IoT applications, such as in healthcare or factories, may require devices to transmit real-time information. RL algorithms have long processing times when dealing with large-scale state–action–reward tuples, which further increases in scenarios where state and action spaces are multi-dimensional [89]. It becomes even more complex when the IoT network is heterogeneous, where some devices are delay-sensitive while others are delay-tolerant. DRL algorithms can be more efficient and quick in learning such complexities; however, running the DRL in tiny IoT devices is quite challenging. In such scenarios, edge computing can be leveraged to offload some of the computation and memory requirements of DRL algorithms. However, transferring a large amount of training data increases the number of overheads and burdens on the IoT networks. Another way to solve such a problem is through transfer learning, in which the agent shares their learned parameters. However, this would require an effective coordination mechanism among the devices, which would increase the convergence delay.

(10) Explorationand Exploitation Performance Trade-off: The exploration–exploitation trade-off involves a decision between selecting already known good actions or choosing actions that are not explored yet. Balancing exploration and exploitation directly impacts the performance of IoT devices. Using an effective exploration strategy such as the upper confidence bound (UCB) algorithm can help the devices to avoid actions that are known to be suboptimal. However, the UCB algorithm requires calculating the confidence bound for each action, which can be computationally expensive. Similarly, stochastic algorithms like UCB usually assume that the probabilities of different outcomes occurring stay the same over time. Such an assumption of *stationarity* can result in the algorithm selecting suboptimal actions. Similarly, UCB also assumes a fixed reward function, which may not hold in IoT applications. In IoT devices, the reward function can be dynamic due to the varying nature of the application requirements or environmental conditions. Similarly, algorithms like Thompson sampling, which can solve the exploration–exploitation dilemma, are susceptible to local optima, particularly when the number of actions is large or the environment is complex. The large action space and complex environment also face an exploration bias problem.

(11) EnergyEfficiency Trade-off in RL-Enabled IoT Environments: RL algorithms require a significant number of samples to effectively learn optimal policies. In IoT networks with limited resources, sample efficiency is crucial to reduce data collection overheads and speed up learning. During learning, the algorithms may involve frequent exchanges of information between nodes, leading to increased communication overheads in IoT networks. Finding lightweight communication mechanisms is essential to minimize energy consumption.

(12) DynamicChanging Environment for RL Algorithms: IoT devices often operate in a non-stationary environment where the underlying distribution of rewards and states can change over time. This leads to poor performance if the environment changes significantly. In such a scenario, *meta-learning* [90] can be useful for an agent to adapt to new environments. Meta-learning also often faces limited generalization problems, i.e., the ability to adapt to a new and unseen scenario. For example, the nodes in IoT networks may join and leave frequently. This dynamic topology can cause instability in RL algorithms, and the routing decisions made by an RL agent may quickly become obsolete as the network topology changes. The quick adaptation to a dynamic changing environment also depends on the sample efficiency.

(13) Implementationin Real-World Scenarios: The primary goal of employing RL algorithms is to optimize the network’s performance, including improving the quality of service, enhancing the network’s energy efficiency, and reducing latency, among other metrics. RL algorithms have proven to be an effective solution for enabling intelligent approaches for IoT device operation and management. However, the majority of current studies solely assess the suggested RL-assisted control strategy through simulations based on random data or models. This approach is far from the practical environment and may not accurately reflect the challenges and complexities of real-world wireless networks. Thus, to enable the widespread deployment of RL-aided schemes in real-world IoT networks, it is necessary to develop robust and efficient algorithms that operate in changing network conditions, such as changes in traffic patterns, user behavior, and device connectivity.

(14) Multi-ObjectiveReward Design Optimization: In heterogeneous IoT networks, devices can have different and multiple performance objectives, e.g., energy efficiency optimization or delay minimization. Thus, designing a practical multi-objective reward function is still a challenge. Devices with different performance objectives need to coexist. Designing a practical multi-objective reward function requires careful consideration of the trade-offs between these objectives. One approach is to assign weights to different objectives which determine their relative importance. However, determining these weights can be challenging, as different devices may have different preferences. One of the ways to find optimal multiple-objective solutions is to use Pareto optimization. However, Pareto optimization can also face the curse of yjr dimensionality problem as the number of objectives increases. With the increase in decision objective variables, the computational complexity of Pareto optimization algorithms can become prohibitively expensive.

(15) Issueswith Enabling FRL: Enabling federated learning is an even bigger challenge for heterogeneous IoT devices with very limited resources. Apart from learning the environment, the devices would need to share their local learning model parameters with other devices, putting a huge burden on a tiny device. Locally computing the learning model and sending the learned model to other devices in a network with a massive number of heterogeneous devices are difficult. Heterogeneity refers to differences in computation resources (i.e., CPU cycles/sec), memory resources, communication, and energy resources. Having heterogeneous parameters can lead to significant differences in learning model accuracy.

## 6. Conclusions

The integration of IoT networks has extended to numerous application areas, making IoT devices ubiquitous in facilitating seamless connectivity and data exchange. Despite the widespread adoption of the IoT, the IoT paradigm also presents a number of challenges, particularly in managing scarce resources and complex networking operations. Traditional standardized protocols, while effective in some scenarios, struggle to cope with the increasing network size and data volume, leading to suboptimal network performance. These challenges stress the need to develop a more intelligent and adaptive approach to unlock the true potential of the IoT paradigm. To this end, machine learning, particularly RL, has been demonstrated as a promising avenue to handle the resource management challenges in IoT networks. This paper highlights the potential of RL for IoT device networking mechanisms. The concept of RL is briefly introduced to emphasize its pivotal role and algorithmic model in facilitating optimal decision making across various practical applications. The objective of this study was accomplished by developing a theoretical framework that serves as a foundational basis for advancing research within the domains of RL and IoT device networking. The primary contribution of the proposed framework is a finer-grained understanding of the strategic decision of the IoT networking layer by adopting an RL algorithm, and while this contribution holds substantial theoretical implications, this study has some limitations that should be addressed in future research. Taking the example of an RPL-based networking layer, this article echoes the call for more research to assess more issues related to the IoT from an RL perspective. The proposed theoretical framework requires additional support through empirical validation.

## Figures and Tables

**Table 1:** List of notations.

Parameters	Labels
*t*	Time step *t*
$s_{t}$	State of the agent at *t*
$a_{t}$	Action of the agent at *t*
$r_{t}$	Reward of the agent at *t*
*A*	Action space
*S*	State space
*R*	Cumulative reward or return
π	Policy
β	Discount factor
α	Learning rate

**Table 2:** Related research studies on the use of RL for network management.

Reference	Year	Contribution	Application Domain	Algorithm Model
Jin et al. [60]	2019	The authors of this paper propose RL-based congestion-avoided routing for underwater acoustic sensor networks to reduce end-to-end delay and energy consumption.	Underwater acoustic sensor networks—RCAR	Q-learning
Di Valerio et al. [61]	2019	In this paper, the authors propose an RL-based data forwarding scheme for a node based on the number of unsuccessful transmissions. The node adaptively switches between single-path and multi-path routing to optimize energy consumption and the packet delivery ratio.	Underwater WSN—CARMA	Q-learning
Safdar Malik et al. [62]	2023	This paper presents a routing approach based on RL for CRs. The idea of this study is to add the channel selection decision capability to provide improvements in the average data rate and throughput.	CRs—RL-IoT	Q-learning
Mao et al. [63]	2019	In this paper, the authors propose a CNN-based scheme that continuously adapts and optimizes routing decisions based on network conditions. This approach computes the routing path combinations with high accuracy.	SDNs	CNN
Safdar et al. [64]	2015	The authors of this paper propose RL-based routing in CR ad hoc networks to reduce the protocol overhead and end-to-end delay and improve the packet delivery ratio.	CRs ad hoc networks—CRAHN	Q-learning
Stampa et al. [65]	2017	This paper proposes a DRL approach for optimizing routing in SDNs. The agent in this approach optimizes the routing policy based on traffic conditions to minimize network delays.	SDN	DQL
Cicioğlu et al. [66]	2023	The authors of this paper proposed an ML-assisted centralized link-state routing system for an SDN-based network. This scheme utilizes historical data of parameters such as the latency, bandwidth, signal-to-noise ratio, and distance to make routing decisions.	SDN—MLaR	Supervised learning
Cheng et al. [67]	2012	In this paper, the authors proposed load balancing in a multi-sink WSN. This approach divides the network into several zones based on the remaining energy of hotspots around the sink node. ML is applied to the mobile anchor, enabling it to adapt to traffic patterns and discover an optimal control policy for its movement.	WSNs—QAZP	Q-learning
Wei et al. [68]	2017	In this approach, the authors present a task scheduling algorithm for dynamic WSNs that minimizes the exchange of cooperative information and balances resource utilization.	WSNs—QS	Q-learning with shared value function
Wei et al. [69]	2019	In this paper, the authors introduce a Q-learning algorithm for task scheduling in WSNs based on support vector machine. Their proposed approach optimizes the application performance and reduces energy consumption.	WSNs—ISVM-Q	Q-learning and support vector machine
Ancillotti et al. [70]	2017	This paper proposes a link quality monitoring strategy for the RPL in IPv6-WSN using a multi-armed bandit algorithm. The proposed approach minimizes overhead and energy consumption by employing both synchronous and asynchronous monitoring.	WSNs—RL-Probe	Multi-armed bandit
Guo et al. [71]	2020	The authors of this paper propose a DRL-based QoS-aware secure routing protocol for the SDN-IoT. The primary objective is to design a routing protocol that efficiently routes traffic in a large-scale SDN.	SDN—DQSP	DQL
Künzel et al. [72]	2020	This paper introduces a Q-learning approach in which an agent adjusts weight values in an industrial WSN, leading to improved communication reliability and reduced network latency.	Industrial WSN—QLRR-WA	Q-learning
Jung et al. [73]	2017	In this paper, the authors introduce Q-learning-based geographic routing to enhance the performance of unmanned robotic networks and address the challenge of network overhead in high-mobility scenarios.	Unmanned robotic networks—QGeo	Q-learning
Sharma et al. [74]	2017	The authors of this paper introduce a tailored Q-learning algorithm for routing in WSNs with a focus on minimizing energy consumption, addressing the challenge of reliance on non-renewable energy sources.	WSNs	Tailored Q-learning
Su et al. [75]	2022	This paper presents an approach to enhance energy efficiency and prolong network lifetime using Q-learning-based routing for WSNs. It allows nodes to select neighboring nodes for transmission by considering various energy consumption factors, resulting in a reduced and balanced energy usage.	WSNs	Q-learning
Akbari et al. [76]	2020	This paper addresses the need for efficient routing structures in sensor networks to optimize their lifetime and reduce energy consumption. The paper combines fuzzy logic and RL, utilizing factors such as the remaining node energy, available bandwidth, and distance to the sink for routing decisions.	WSNs	RL with fuzzy logic
Liu et al. [77]	2019	The authors of this paper address the importance of connectivity solutions for wide-area applications in IoT networks. The proposed technique uses a distributed and energy-efficient RL-based routing algorithm for wide-area scenarios.	Wireless mesh IoT networks	Temporal difference
Sharma et al. [78]	2020	In this paper, the authors propose routing in opportunistic IoT networks using the Policy Iteration algorithm to automate routing and enhance message delivery possibilities.	IoT networks—RLProph	Policy Iteration algorithm
Chakraborty et al. [79]	2022	In this paper, the authors proposed a routing algorithm that adjusts its routing policy based on local information, aiming to find an optimal solution that balances the network latency and lifetime in wireless mesh IoT networks.	Wireless mesh IoT networks	Q-learning
Muthanna et al. [80]	2022	This paper presents a system that optimizes transmission policy parameters and implements multi-hop routing for a high QoS in LoRa networks.	LoRa IoT networks—MQ-LoRa	Soft actor-critic
Kaur et al. [81]	2021	The authors of this paper proposed an algorithm that divides the network into clusters based on sensor node data loads, preventing premature network failure. This paper addresses issues such as high communication delays, low throughputs, and poor network lifetimes.	IoT-enabled WSNs	DQL
Zhang et al. [82]	2021	The authors of this paper use recurrent neural networks and the deep deterministic policy gradient method to predict the network traffic distribution. They employ a double deep Q-network to make routing decisions based on the current network state.	IoT-enabled WSNs	RNN and the deep deterministic policy gradient
Krishnan et al. [83]	2021	This paper focuses on addressing the challenge of maximizing the network lifetime in WSNs. Q-learning is employed to facilitate automatic learning to find the shortest routes.	IoT-enabled WSNs	Q-learning
Serhani et al. [84]	2020	This paper explores the challenges of integrating MANETs with the IoT and focuses on the issue of network node mobility. The authors introduce an adaptive routing protocol that enhances link stability in both static and mobile scenarios.	MANETs-IoT systems—AQ-Routing	Q-learning
Pandey et al. [85]	2022	In this paper, the authors address the challenge of establishing large-scale connectivity among IoT devices. They introduce a multi-hop data routing approach utilizing the Q-learning method.	Low-power wide-area networks for IoT	Q-learning
Ren et al. [86]	2023	In this paper, the authors address the challenges of energy efficiency and network lifetime using the mean field RL method. Mean field theory simplifies interactions among nodes, and a prioritized sampling, loop-free algorithm prevents routing loops.	IoT-enabled WSNs	Mean field RL
Serhani et al. [87]	2023	In this paper, the authors introduce an efficient routing mechanism for the Internet of Medical Things. The proposed technique categorizes network traffic into three classes, optimizes paths based on QoS and energy metrics, and employs RL for path computation.	Internet of Medical Things—EQRSRL	Q-learning

## Abbreviations

The following abbreviations are used in this manuscript:

IoT	Internet of Things
RL	Reinforcement learning
LLN	Low-power and lossy networks
MAC	Medium access control
RPL	Routing protocol for low-power and lossy networks
DODAG	Destination-oriented directed acyclic graph
BE	Backoff exponent
ML	Machine learning
CRs	Cognitive radios
LoRa	Long-range
DRL	Deep learning with RL
OF	Objective function
OF0	Objective function zero
MRHOF	Minimum rank with hysteresis OF
ETX	Expected transmission count
DIO	DODAG information object
DAO	DODAG advertisement object
DIS	DODAG information solicitation
DCF	Distributed coordinated function
DIFS	DCF interframe space
CW	Contention window
NB	Number of backoffs
ACKs	Acknowledgments
MDP	Markov decision process
RCAR	RL-based congestion-avoided routing
CARMA	Channel-aware RL-based multi-path adaptive routing
ISVM-Q	Q-learning-based improved support vector machine
QLRR-WA	Q-learning reliable routing approach with a weighting agent
SDNs	Software-defined networks
CNNs	Convolutional neural networks
QAZP	Q-learning-based adaptive zone partition
WSNs	Wireless sensor networks
MANETs	Mobile ad hoc networks
MiFi	Mean field RL
UCB	Upper confidence bound
